# Identifying determinants of viral hepatitis and liver cancer care in Michigan Asian American communities through multilevel engagement

**DOI:** 10.1097/HC9.0000000000000803

**Published:** 2025-09-05

**Authors:** Parnnate Wongsirisakul, Neehar Parikh, Yi-Chun Wang, Hannah Par, Thanvir Chowdhury, Qingqing Zhang, Tsu-Yin Wu, Ponni V. Perumalswami

**Affiliations:** 1 Division of Gastroenterology and Hepatology, University of Michigan, Ann Arbor, Michigan, USA; 2 School of Public Health, University of Michigan, Ann Arbor, Michigan, USA; 3 Center for Health Disparities Innovations and Studies, Eastern Michigan University, Ypsilanti, Michigan, USA

**Keywords:** Asian American, HBV, HCV, liver cancer, viral hepatitis

## Abstract

**Background:**

In Michigan, Asian Americans are disproportionately infected with HBV and HCV. As many infections are first diagnosed when patients present with advanced liver disease or liver cancer, HBV and HCV screening, awareness, and early treatment are critical to improving outcomes.

**Methods:**

Using a theory-informed approach, we administered a bi-level qualitative study to identify determinants of viral hepatitis and liver cancer care and treatment in Michigan Asian American communities. We conducted a focus group involving representatives from public health agencies, cancer coalitions, and Asian diaspora organizations across the state. We then completed 1:1 interviews with leaders from the communities. Groups and interviews were taped, transcribed, and used to identify common and distinct themes according to the National Institute of Minority Health and Health Disparities framework.

**Results:**

According to community leaders, language barriers, costs, and a lack of viral hepatitis education appeared among the top shared screening barriers between the 3 communities. Conversely, common facilitators included pre-existing health programs, interpretation services, and community partnerships. Such sentiments were also echoed by the stakeholder focus group. However, the communities also raised distinct concerns about medical mistrust and positive health messaging.

**Conclusions:**

This qualitative report marks the first phase of a bi-level mixed methods study in Asian American Michigan communities to understand determinants of viral hepatitis and liver cancer care and treatment. This work lays the foundation for a quantitative survey that will gather data from community members to inform the development of a future intervention.

## INTRODUCTION

Viral hepatitis B and C (HBV and HCV) infections are leading causes of cirrhosis and HCC, the third most common cause of cancer-related death worldwide.^1^ HCC surveillance is recommended in some people living with HBV and in people living with HCV cirrhosis.^2^ Lack of recognition of underlying liver disease, including viral hepatitis, is a substantial risk factor for presentation with advanced-stage HCC. Early detection of HCC is associated with a significantly higher 5-year survival rate (70%) versus detection during advanced progression (<5%).^3^^,^^4^ There are several available treatments to cure HCV or suppress HBV, which decrease the risk of disease progression and the development of advanced liver disease and HCC. Because infections are often asymptomatic, early detection relies on screening. Therefore, improvements in screening for HBV and HCV could result in millions of life-years saved annually.^5^ Without active screening, many viral hepatitis infections are first diagnosed when patients present with advanced liver disease or liver cancer, thus highlighting the need for HBV and HCV awareness, screening, and early treatment.^6^

In the United States, <20% of people with known infection receive follow-up care. Up to 70% of persons infected with HBV living in the United States are foreign-born, and 1 in 12 Asian Americans/Pacific Islanders (AAPI) are chronically infected with HBV.^7^ AAPI make up more than 7% of the total population in the United States but account for 60% of the 862,000 Americans living with HBV.^8^^,^^9^ In Michigan, based on 2020 data, AAPI have a substantively higher HBV infection rate of 32.86 per 100,000, compared with the state average of 10.93.^10^ Similarly, HCV infection has been shown to be prevalent in certain immigrant communities from countries where HCV is endemic (>2% prevalence), such as South and East Asia.^11^^,^^12^ Furthermore, liver cancer in Michigan has increased by 50% between 2007 and 2016 and is now the sixth leading cause of cancer death in the state, with half of the cases being caused by HCV infection. It is now recommended that all US adults receive at least 1 HBV and HCV test in their lifetime.^13^ Viral hepatitis screening, treatment, and linkage to care remain a key cancer control strategy to prevent the development of HCC, especially among traditionally underserved populations.

The current literature on viral hepatitis screening in AAPI immigrants in the United States does well to provide a sociocultural context for the biomedical statistics. Survey studies on various AAPI groups (Chinese, Cambodian, Korean, Hmong, and Vietnamese) in different states bring to light gaps in hepatitis and liver cancer knowledge, as well as unique stigmas and beliefs that act as barriers to screening.^14^^–^^18^ Although such research has provided us with greater insights into AAPI rationale, data collected solely from individual community members do not offer insight into the diaspora of communities as a whole. As a result of the lack of multilevel engagement, there is no framework to promote screening and future collaborative activities. Thus, it is crucial to understand how stakeholders, community leaders, and community members interact to bridge gaps in communication and goals and create feasible action plans. We aimed to characterize determinants of viral hepatitis and liver cancer awareness and care through engagement of a multilevel stakeholder panel in 3 immigrant communities in Michigan. These communities not only differ culturally but also structurally, as each community is located in areas with varying degrees of urbanization.

## METHODS

The overarching goals of this implementation science project, REducing AAPI Community Health disparities for LIVER Cancer in MIchigan (REACH-LIVER MI), are to collaborate with key Michigan stakeholders to reduce liver cancer disparities in AAPIs at-risk for HBV and HCV in the University of Michigan’s Rogel Cancer Center catchment area and to assess viral hepatitis and liver cancer prevention and control needs. We used a qualitative, bi-level design that included a focus group with external stakeholders, followed by 1:1 interviews with community leaders from the 3 AAPI communities. Findings from the qualitative studies will be used to inform the development of a community participant survey to assess determinants of viral hepatitis and liver cancer testing and care in AAPI communities. We gave external stakeholders and community leaders the option to meet in person versus conduct meetings virtually. All meetings were held over video conference and moderated primarily by trained study staff and the principal investigator, with 1 or 2 members of the research team taking notes and monitoring the recording. Upon completion, each participant received a gift card incentive.

**FIGURE 1:**
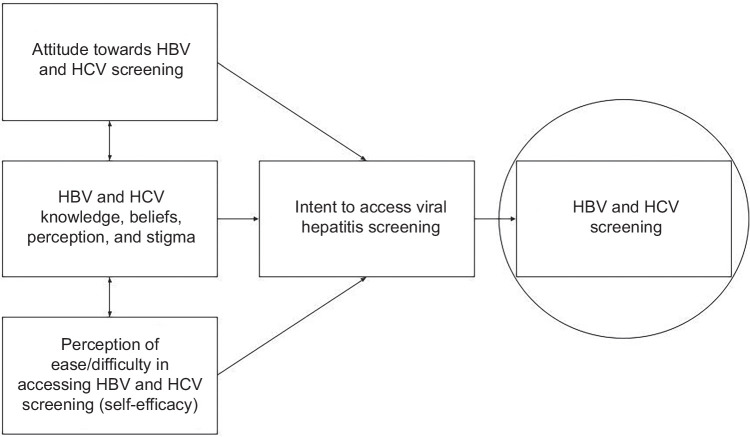
Theory of Planned Behavior for viral hepatitis screening, adapted from Ajzen et al.^19^

The questions used to guide the stakeholder focus group and the subsequent community leader interviews (see Supplement 2, http://links.lww.com/HC9/C119) were inspired by the Theory of Planned Behavior (TPB, Figure 1). TPB predicts individual intention to engage in a behavior.^20^ Behavioral achievement (ie, viral hepatitis and liver cancer screening) depends on motivation (or intent) and ability. Our previous research with the Hepatitis Outreach Network (HONE)^21^ in West African communities in New York City focused on distinguishing sociocultural constructs to understand viral hepatitis screening behaviors in underserved minority and immigrant communities by adapting the TPB.^22^^–^^24^ By linking one’s beliefs to behavior, TPB has predicted health behaviors, including HIV testing and cancer screening.^25^^–^^29^ Key TPB constructs to be assessed include: (1) attitudes toward screening; (2) perceived subjective norm to assess knowledge, beliefs, perceptions, and stigma; and (3) perceived behavioral control to get screened, or perceived ease or difficulty in performing the behavior.^28^

### Ethical compliance

All research activities were approved and reviewed by the University of Michigan Institutional Review Board before study initiation. All research was conducted in accordance with both the Declarations of Helsinki and Istanbul. Due to the data collection method and lack of sensitive data, this study was exempted by the Institutional Review Board. Although written consent was waived, participants were informed of the nature of the study and verbally consented before the start of focus groups and interviews.

### Data collection

**FIGURE 2:**
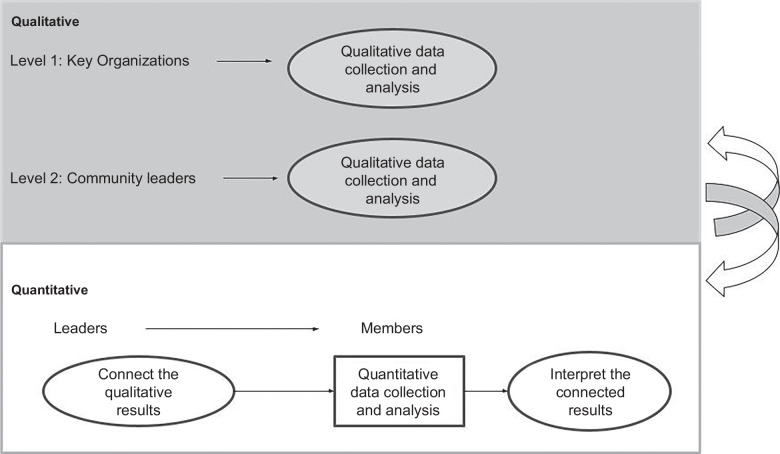
Bi-level mixed methods.

Level 1 of REACH-LIVER MI’s bi-level design (see Figure 2, with this report’s current focus highlighted in gray) invited 8 external stakeholders (see participating organizations in Supplemental Table S1, http://links.lww.com/HC9/C118, found before references) from across the state, representing payers, cancer coalitions, community health workers, the Michigan Department of Health and Human Services (MDHHS), and Asian diaspora associations. Together, we conducted a 2-hour-long focus group with the goal of identifying broad cancer (liver and non-liver) and viral hepatitis screening behaviors and linking them to care determinants for Michigan AAPI communities. After its completion, we began collaborating with community leaders for the second level of the study.

Level 2 consisted of 1:1, semi-structured, hour-long interviews (n=3–5/community) with 12 leaders from 3 pre-selected Michigan AAPI communities—the Chinese community in Detroit, MI, the Bangladeshi community in Hamtramck, MI, and the Burmese community in West Michigan. The leaders were selected through snowball sampling and contacted by email and phone, beginning with recruitment efforts from Eastern Michigan University’s Center of Health Disparities Innovations and Studies (CHDIS). The target communities were identified by Asian Communities Toward Innovative Visionary Environment (ACTIVE) based on their high hepatitis rates and underutilization of preventive health services in pilot research conducted by Dr Tsu-Yin Wu (Co-PI, CHDIS).

Our interviews evaluated (1) general cancer screening practices in their community; (2) viral hepatitis and liver cancer knowledge, attitudes, and beliefs; (3) health and health care access; and (4) best practices for community engagement. The focus group and the following community leader interviews were facilitated by the Principal Investigator (P.V.P.) and Co-Investigator (N.P.)—both physicians—alongside a research coordinator with a bachelor’s degree in public health (Y.C.W.). Before the study started, the interviewers had no previous relationships with the individuals interviewed. Interviews were conducted in English, audiotaped, and transcribed for detailed analyses that may guide future implementation.

### Analysis

Focus group and interview transcripts were independently reviewed by a research coordinator (Y.C.W.) trained in qualitative research methods and the Principal Investigator (P.V.P.), then compared against one another to ensure rigor in coding. Analyses were completed using a rapid data analysis process—a qualitative approach used with time-sensitive, targeted, and actionable qualitative information^30^—to group the apparent themes according to the National Institute of Minority Health and Health Disparities^30^ (NIMHD) and TPB frameworks. A detailed breakdown of how the themes were coded is included in Supplement 3, http://links.lww.com/HC9/C120.

## RESULTS

A table summarizing interview participant details and overall opinions on viral hepatitis is included below (Table 1). A “moderate” rating for knowledge of viral hepatitis and liver cancer was noted if community leaders believed members of their community were familiar with these terms but were unsure of details such as modes of transmission, severity, screening, etc. If few community members had heard of these conditions previously, a “low” rating was assigned. In regard to community and community leader support of HBV/HCV prevention, both Bangladeshi and Chinese leaders expressed that members would gladly partake in any organized programs, and the leaders would be eager to lend their efforts, thus resulting in a “high” support rating. However, the Burmese community had a “moderate” rating because members were described as interested in health programs but unlikely to set aside time to attend. Similarly, Burmese leaders were willing to endorse prevention efforts but explained that they were already overwhelmed by planning numerous community events.

**TABLE 1:** Community leader involvement and opinions on HBV/HCV knowledge and intervention

AAPI community	Bangladeshi	Chinese	Burmese
Number of leaders interviewed	4	5	3
Position within their affiliated AAPI organization	Full-time employees	Split between volunteers and full-time employees	Mostly full-time employees
Avg time spent working w/ AAPI community	5 years	9 years	5 years
HBV/HCV knowledge among the community	Low	Moderate	Low
Liver cancer knowledge among the community	Low	Moderate	Low
Community leader support toward HBV/HCV prevention	High	High	Moderate
Community support toward HBV/HCV prevention	High	High	Moderate
Most useful ways to promote health information	Religious organizations, social media	Social media, flyers	Religious organizations, social media

**FIGURE 3:**
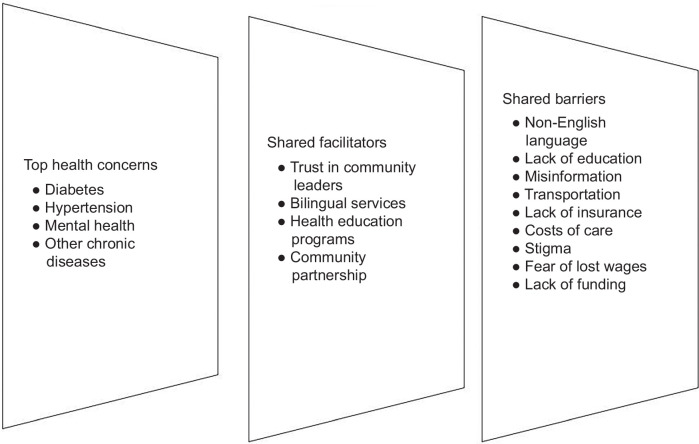
Shared facilitators and barriers to hepatitis testing as indicated by community leaders.

**FIGURE 4:**
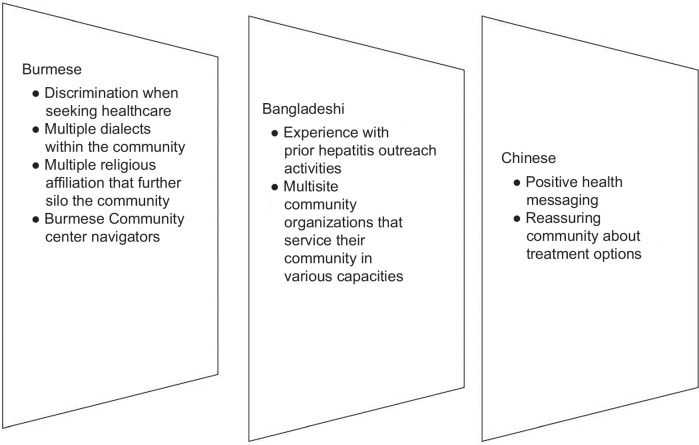
Distinct facilitators and barriers to hepatitis testing as indicated by community leaders.

Amid the feedback from external stakeholders and community leaders, 5 common themes emerged: physical/built environment, sociocultural environment, health care system, knowledge, and health attitudes and beliefs. The first 3 themes listed fall under the National Institute of Minority Health and Health Disparities framework, while the last 2 follow the TPB. Each theme was then further divided into its “facilitator” and “barrier” aspects. Table 2 illustrates the facilitators and barriers according to the perspectives of external stakeholders, whereas Figures 3 and 4 depict shared and distinct facilitators and barriers according to the community leaders. Within the barrier domain, one unique factor, costs/funding, was also apparent. External stakeholders highlighted how policy restrictions, such as prior authorization for viral hepatitis screenings, especially in Medicaid-holders, can act as a major deterrent. Across all 3 AAPI communities, leaders expressed their wishes to partner with more local and federal organizations like the MDHHS to receive the funding and resources needed to support awareness campaigns tailored to communities.

**TABLE 2:** Facilitators and barriers to hepatitis testing as indicated by external stakeholders

Facilitators	Barriers
Applicable across all Michigan communities	
Awareness/education campaigns	Cost/funding
Michigan HCV elimination plans	Work
Financial navigation	Lack of knowledge
Specific to Michigan Asian American communities	
Community engagement	Language barriers
Community navigators	Cultural barriers
Convenience of testing/care	Transportation

### Physical/built Environment

#### Transportation

Within the theme of physical/built environment, most community leaders and external stakeholders identified the lack of transportation as a common barrier that may prevent people from utilizing their local screening locations. Leaders expressed the potential difficulty of understanding and asking about the bus schedule for community members who struggle with English. Particularly for the Burmese community in West Michigan, one leader noted that ride-sharing services (eg, Uber and Lyft) are rarely seen in West Michigan.

#### Work priorities

Aside from transportation, there was a consensus on the restrictiveness of work priorities, especially for communities with younger age demographics, such as the Bangladeshi and the Burmese communities. Community members fear losing wages or employment if they were to ask for time off to get tested. This fear is further compounded by the possibility of a positive blood test for an infection, which would result in taking even more time off to handle health matters. The imbalance in work-health value was evident in the words of one Bangladeshi leader:

Suppose he earn $1, instead, he said, “Oh, I earn 100 Takas” in our currency. So, they try to concentrate more of their efforts to work more. They don’t care about their health.

One way to address scheduling conflicts and transportation issues is to meet people where they are. Fortunately, each community participates in some form of large-scale gathering that can be coupled with HBV/HCV educational promotions and potential screening. In the Bangladeshi community, many are members of the same mosque in which their religious leader, the Imam, reserves a period before prayer to bring attention to community updates. Meanwhile, the major ethnic group in the West Michigan Burmese community is the Chin, and they hold annual celebrations for Chin New Year and Chin National Day. The Chinese community has similar cultural events and often assembles for food festivals, where the leaders already set up booths to promote ongoing community health programs. If we were to table at one of these events, we would need to develop educational flyers and create an interactive display. One Chinese leader recommended that we integrate viral hepatitis knowledge questions into a game-like format, in which families could test their knowledge and win prizes. A list of other community leader-indicated barriers to viral hepatitis testing and their corresponding proposed interventions can be found in Table 3.

**TABLE 3:** Proposed interventions for barriers to viral hepatitis testing

Barrier	Intervention
Lack of funding for community health programming	Partnering with local and federal organizations like the Michigan Department of Health and Human Services
Scheduling conflicts	Promoting prevention efforts at community celebrations or religious gatherings
Medical mistrust	Using a train-the-trainer model to teach community leaders how to deliver educational materials, or inviting culturally similar health professionals to deliver the education
Access to support and language concerns	Connecting to resources (ie, translation services) through social media
Lack of knowledge, stigma, and fear of infection	Developing a viral hepatitis educational program that focuses on destigmatizing infection and explaining treatment in a hopeful manner
Transportation	Reimbursing participants for travel expenses to vaccination/screening centers
Motivation	Targeting educational interventions to middle-aged members of the community
Costs of care	Including state-wide health initiatives and health insurance coverage details in the education

### Sociocultural environment

#### Language barriers

Every interview revealed a strong agreement on language as the main barrier to screening. However, of the 3 communities, the Burmese community experienced the most hindrances with regard to sociocultural environment. Several different dialects divide the Burmese community, thereby limiting the reach of health programs.

#### Medical mistrust, racism, and discrimination

In addition to obstacles in communication, the leaders described a high level of medical mistrust, rooted in a fear of discrimination and prior negative encounters in the health care system, as recounted by one participant who works as a Tedim (a Burmese dialect) medical interpreter:

Even though our parents don’t understand the language, they can tell when they’re [medical staff] being racist, and they don’t feel comfortable going to that hospital. They’re like, “Oh, man! The way that he or she talking to me…they belittle me.” I would hear a lot of that.

#### Unsupported age groups

According to the Chinese and Burmese leaders, their elderly populations were especially vulnerable. Seniors tended to isolate themselves as they believed that asking for help (ie, rides to the hospital or a grocery store) would burden their family members. One leader explained:

“Yes, it’s just like in Chinese saying that 水只向下流,不往上” [The water only goes down, meaning that only the parents give and contribute to the children. It does not go both ways.]

Despite these challenges, each community had established a framework to support its members. Translation and navigation services are widely offered, and those who want to use the services or have inquiries can easily connect through Facebook groups and other social media platforms. Furthermore, community members placed an immense amount of trust in their leaders, who are outreach-focused, so it would be best to integrate these community leaders into future intervention implementation. A train-the-trainer model could possibly be used to turn community leaders into lay health workers who can present viral hepatitis educational materials.

### Health care system

#### Costs and health insurance issues

Under the health care system, lack of insurance and lack of knowledge on insurance coverage were the most prevalent. Specifically, leaders shared that among undocumented refugees—which are the more recent immigrants in the Burmese community—many, if not all, do not qualify for health insurance, thus presenting a major barrier to accessing care. In addition, many AAPI are employed in service industry and manufacturing positions, such as restaurant and factory work, that either do not provide health insurance, pay a sufficient salary to cover the expenses of the US medical system, or are too costly compared with time away from work to pay for medical expenses.

#### Medical beliefs and behaviors

For the most part, many leaders across the communities felt the high cost of treatment in the United States drives immigrants to prefer to seek care in their home countries, but cultural beliefs also play a role. During the interviews, Chinese leaders discussed how it was not uncommon for community members to opt for traditional over Western medicine due to a greater perceived effectiveness of traditional remedies. Some even equate Western medicine to “chemicals” and believe it will worsen their conditions.

When community members do engage with the US health care system, it is prompted by persistent symptoms that alter their daily routines, and the treatment they receive is often incomplete due to the cost of follow-up care. In relation to this kind of symptom-driven health-seeking behavior, one Burmese leader commented:

If it’s just like a pain that stops, that’s fine, they would not probably go. But if it’s like hurting, and it wouldn’t stop, I think that’s when they would go, which sounds like it would be too late.

These words capture the reality of the AAPI population served by the Rogel Cancer Center, who oftentimes begin treatment only after they discover how much their liver diseases have progressed. To motivate people to engage in prevention and early detection over late-stage intervention, community leaders suggested offering compensation for costs related to vaccination and screening. For example, past studies, including the HONE project, have reimbursed participants for travel-related expenses to the testing center.^21^^,^^32^

### Knowledge

#### Misinformation and lack of information

Throughout the interviews, HBV/HCV and liver cancer misinformation was apparent among the communities and the community leaders. The exception was the Bangladeshi leaders, who were informed of HBV and HCV transmission routes because of prior work in viral hepatitis education. When other leaders were asked about their prior HBV and HCV knowledge, sentiments like *“*I don’t need the vaccine. I don’t drink alcohol” and “sharing bowls and chopsticks can spread HBV” came up—thus revealing a need for an awareness-centered approach to screening. In addition to a better understanding of viral hepatitis transmission, symptoms, and outcomes, Bangladeshi leaders called for more awareness on the part of primary care doctors. They felt as though their local doctors were not advising their patients to get tested for HBV and HCV, which is problematic as all leaders described their community populations as reserved around medical staff. Community members noted reluctance to initiate dialogue with their providers due to language barriers, low health literacy, and/or cultural norms. For example, a Burmese leader mentioned the rigidity of the doctor-patient relationship in Myanmar:

You’re not supposed to look at, you know, people of authority, like for example, doctors…I never looked them in their eyes, because that’s what I was taught. That’s a sign of disrespect.

#### Pre-existing health initiatives

In contrast, the familiarity of pre-existing community health programs—viral hepatitis and non-hepatitis–related—are facets of the knowledge theme that can help facilitate education and testing efforts. All 3 communities offered courses as part of the Diabetes Prevention Program, occasional general wellness checks (blood sugar, blood pressure, and BMI), and flu and COVID vaccination clinics—all of which were extremely popular. In terms of cancer screening, the Bangladeshi community was unique in its history of stool test dissemination for colorectal cancer and its partnership with CHDIS to obtain a mobile mammography van for the community. Under the guidance of the Hepatitis B Foundation, one Bangladeshi leader even conducted a limited survey study on HBV awareness in his community, which concluded that Bangladeshi knowledge and testing behaviors were very low. The Chinese leaders also reported providing liver and colon cancer screenings in the early years of their organization in community settings, but a lack of funding made it difficult to sustain. In their more recent years, Chinese leaders collaborated with a local Asian Pacific American Medical Student Association (APAMSA) to conduct HBV educational workshops and blood tests in the Chinese community, but the turnout was much lower than other community health programs that target many health issues more broadly.

### Health attitudes and beliefs

#### Stigma and confidentiality

Under health attitudes and beliefs, 3 subthemes emerged from the transcripts: stigma, fear of testing positive for infection, and aversion to outsiders. Leaders describe these 3 factors as being intrinsically linked, as many Asian Americans worry that their lab results will be disclosed by unfamiliar providers to their communities, thereby causing stigma among friends and families. To increase viral hepatitis testing efforts, a key factor would be to emphasize patient privacy with test results. Based on the interview responses, serious illnesses are typically stigmatized. For those who do not understand disease transmission, the afflicted person may be avoided in a misguided attempt to stop the spread of said disease. As one leader stated:

It’s very common for the Chinese, if the husband got sick or the wife got sick, they just shut the door. They don’t even want to see their friends.

#### Fear of positive infection

Lab results can also be accompanied by a sense of dread. One community leader noted that a positive HBV or HCV infection can make people feel as if they were “cursed.” They are afraid of their prognosis and worry that they will burden their families. However, by framing hepatitis education and the steps following screening in a hopeful light, more Asian Americans would be willing to find out their status. In addition to disseminating positive health messaging, education should be destigmatized. Some leaders suggested presenting personal connections (ie, testimonials from celebrities with viral hepatitis or liver cancer) to cultivate an accepting environment.

## DISCUSSION

Known determinants of low viral hepatitis screening in AAPI and other immigrant communities based on our past work and those of colleagues include the asymptomatic nature of most viral hepatitis infection, lack of access to accurate viral hepatitis information, cultural beliefs around health-seeking behavior (symptom-driven vs. prevention/early detection), lack of insurance, difficulties navigating into health practices and systems, limited English proficiency, and low health literacy.^8^^,^^33^^–^^36^ However, the conclusions made from these publications cannot be extrapolated to other populations. Michigan is a geographically different area from cities such as New York, and the communities at-risk, along with their determinants, are distinct. Thus, we sought to conduct a separate study to examine common and unique determinants of viral hepatitis and liver cancer testing and care across the Michigan Bangladeshi, Chinese, and Burmese communities. Through a focus group of 8 external stakeholders and interviews with 12 AAPI community leaders, we explored how barriers and facilitators in the physical/built environment, sociocultural environment, health care system, knowledge, and health attitudes and beliefs affect Asian American access to HBV and HCV screening and care.

External stakeholders and community leaders alike identified pre-existing health programs as facilitators of viral hepatitis testing. Program attendance shows a history of health-minded interest within the communities, and it is suggested that individuals who have previously participated in cancer screening may be more willing to partake in other preventive health measures.^37^ Furthermore, these programs—along with the widespread network of local AAPI organizational outreach—can be used to advertise state-wide health initiatives for those who are apprehensive about testing and treatment costs and coverage. Specifically, the MDHSS began the We Treat Hep C initiative as a part of Michigan’s State Plan on Eliminating Hepatitis C in 2021. The project significantly lowers the cost of HCV screening and medications—especially for patients on Medicaid^38^—which comprises a large portion of target community members.

With regard to barriers, stakeholders and community leaders agreed on the limiting role of misinformation and stigma. The lack of viral hepatitis awareness and education substantially impacts the desire of community members to understand their testing status because they are unaware of their chances of infection and the severity of potential disease progression. Community leaders posit that if we conveyed the importance of screening, people would be more likely to rearrange their schedules to get tested and follow up. However, it is essential that the education we provide not only communicates the risks but does so in a way that can alleviate stigma. In our previous project, West African emigrants reported that much of the stigma surrounding viral hepatitis was rooted in the possibility of transmission through unprotected sexual intercourse.^39^ To tailor the education to Michigan Asian American community knowledge gaps and identify areas of bias, we will include questions pertaining to stigma in the future community member survey.

While the community leaders all concluded that hepatitis and liver cancer education would motivate more people to prioritize their health, the role of family seemed to vary. Families may reinforce stigma, but Chinese community leaders explained that if their family members get tested for hepatitis, this is likely to reduce stigma and lead to other family members getting tested. This trend is consistent with findings from other Chinese immigrant communities that show how families can decrease stigma and positively influence health behaviors.^16^ In self-reported close-knit communities like the Burmese and the Bangladeshi, leaders vocalized that the desire to help one another would eventually overcome stigma. Furthermore, communities with strong social ties tend to have more members with feelings of moral obligation toward the group. These members are more likely to take initiative in knowing their screening status to protect others, which aligns with the TPB’s consideration of personal norms as an addition to subjective norms.

Another factor that differed between communities was the extent of each community’s experience with racism and medical mistrust. Although all leaders expressed a shared discomfort in interacting with health care providers, the Burmese leaders spoke most about personal and second-hand accounts of negative encounters while seeking treatment. This contrast may be related to the absence of local, culturally similar doctors in West Michigan. When asked whether their community members concentrated their attendance at a particular medical practice, Bangladeshi and Chinese leaders replied that they patronized providers who could understand their perspectives. Members of the Chinese community gravitated toward Chinese doctors, and members of the Bangladeshi community sought out Indian and Pakistani doctors since there are few Bangladeshi providers in the Hamtramck area.

Age-related priorities mark an additional distinction between the 3 communities. Communities comprised of younger members, on average, such as the Burmese and Bangladeshi, assigned more importance to earning income over maintaining their health if the problem was not urgent. Between their jobs and familial obligations, they struggle to allot time to attend check-ups and community welfare events. In contrast, the higher proportion of retired members in the Chinese community was described as having abundant free time but little interest. According to Chinese leaders, their older members would rather garden or participate in other relaxing activities instead of stressing about their health. With these considerations, we should target educational interventions toward the middle-aged group of each community, as community leaders explained that elderly members would be more receptive toward their children, and young adults would be more receptive toward their parents.

Following the conclusion of this qualitative study, we plan to use this collection of responses—along with an approach guided by the TPB—to inform the next step of a bi-level, mixed methods design, which entails drafting and distributing a survey to community members to create a quantitative profile of each community’s viral hepatitis awareness and practices. Perceived behavioral control, otherwise known as self-efficacy, refers to confidence in one’s ability to achieve the behavior of interest. It is strongly influenced by *actual* behavioral control, which is dependent on available resources and opportunities. According to the interview transcripts, screening costs, transportation, language disconnects, and health care system navigation were among the greatest concerns of the community leaders and stakeholders. Thus, we will focus on these topics in the survey’s self-efficacy section. In addition to these structural barriers, we will consider community members’ experiences with racial discrimination and group-based medical mistrust as they relate to perceptions of ease/difficulty in accessing care.

## CONCLUSIONS

By utilizing a theory-informed approach and applying a health disparity framework, we qualitatively examined Michigan AAPI community perceptions of their physical/built environment, sociocultural environment, health care system, knowledge, and health attitudes and beliefs in relation to HBV and HCV services. The results emphasize the importance of increasing HBV and HCV education and opportunities to access care in a manner that is culturally competent and destigmatized. The community leaders and external stakeholders also suggest collaborating with religious organizations, disseminating information through social media, and addressing fears of a negative disease outlook. Hesitancy regarding screening costs and health insurance was also evident in the discussions. Altogether, this feedback creates the foundation for a comprehensive survey that will allow community members to elaborate on their unique needs as we move to bridge gaps in hepatitis care and control.

## Supplementary Material
