# Supplementary material for: Identifying determinants of viral hepatitis and liver cancer care in Michigan Asian American communities through multilevel engagement
Source: Hepatol Commun. 2025 Sep 5;9(9):e0803. doi: 10.1097/HC9.0000000000000803 (PMC12412735; doi:10.1097/HC9.0000000000000803)
Supplement: Supplementary file 1 [file hc9-9-e0803-s001.docx]

**Supplement 1: List of Key Stakeholders**

| Rogel Cancer Center (Rogel) | The Rogel catchment area was expanded in 2020 to cover the entire state of Michigan which is comprised of ~10 million residents (74.7% White, 14.1% Black, 5.3% Hispanic, 3.4% Asian). Liver cancer is a Rogel cancer priority with greater health disparities in minorities with incidence and mortality seen in Rogel catchment area than in the US. Dr. Neehar Parikh (Co-PI) is an active member of Rogel Cancer Center and the Cancer Disparities work group. |
| --- | --- |
| Asian Communities Toward Innovative Visionary Environment (ACTIVE) | State-wide coalition including state and federal legislators, the Michigan Department of Health and Human Services (MDHHS), city and county public health departments, and AAPI leaders that provides strategic support to Eastern Michigan University Center for Health Disparities Innovation and Studies (CHDIS) led by Co-PI, Dr. Tsu-Yin Wu. ACTIVE has representation from Chinese, Bangladeshi, and Burmese communities and the coalition will help identify community leaders of the target communities. |
| Michigan Cancer Coalition (MCC) | Statewide cancer coalition that operates as a partnership between public and private organizations to provide a forum for collaboration to reduce the burden of cancer among Michigan citizens. Through collaboration with the MCC, we strive to contribute to two priorities of the Michigan Cancer Plan (2021-2030)—1) increasing the number of cases who achieve HCV cure and 2) increasing the percentage of MCC organizations and partnerships that represent underserved communities/populations. |
| Michigan Community Health Workers Alliance (MiCHWA) | Leader in training, certification, and advocacy for CHWs in Michigan. Their mission is to promote and sustain the integration of CHWs into Michigan’s health systems through policy and workforce development. MiCHWA has partnered with the University of Michigan in various capacities and is planning work to engage and train CHWs in the three target AAPI communities. |
| Michigan Department of Health and Human Services (MDHHS) Viral Hepatitis Unit | The MDHHS Viral Hepatitis Unit has strongly supported integrated viral hepatitis screening efforts since its inception in 2013. With the launch of a statewide HCV Elimination Plan in April 2021, barriers to HCV treatment have been reduced to increase treatment. They also support HBV testing and treatment and issue an annual report on viral hepatitis and liver cancer, which highlights the burden of disease particularly in underserved immigrant communities. |
| MDHHS Cancer Section | MDHHS Cancer section aims to reduce cancer incidence and mortality by promoting prevention, early detection, treatment, and caring for those diagnosed using public health approaches. Addressing cancer disparities and promoting health equity is vital in this work. |
| MDHHS Medicaid | MDHHS Medicaid has been involved in HCV Elimination efforts and removing barriers to HCV treatment and cure. Michigan Medicaid is now one of 11 states in the US that does not require prior authorization for HCV treatment. |
