# Supplementary material for: Identifying determinants of viral hepatitis and liver cancer care in Michigan Asian American communities through multilevel engagement
Source: Hepatol Commun. 2025 Sep 5;9(9):e0803. doi: 10.1097/HC9.0000000000000803 (PMC12412735; doi:10.1097/HC9.0000000000000803)
Supplement: Supplementary file 2 [file hc9-9-e0803-s002.docx]

**Supplement 2: Interview Guide**

1. In your opinion, what are the main health concerns for Michigan AAPI communities?
   1. Prompts:
      1. What are the top three concerns?
      2. How much of a concern is viral hepatitis and cancer prevention in Michigan Asian American Pacific Islander communities?
2. What are the main factors that may influence individuals to seek care in Michigan Asian American communities?
   1. Positive factors:
   2. Negative factors:
3. What are the main factors that influence cancer screening in the Michigan AAPI community?
   1. Factors that promote viral hepatitis and cancer screening?
   2. Factors that impede viral hepatitis cancer screening?
4. How do people in Michigan feel about community health worker-led outreach programs?
   1. Are there currently community health worker-led cancer prevention or outreach programs in Michigan?
      1. If yes, which communities utilize this type of model the most?
      2. If not, why do you think that is the case?
   2. Are there currently community health worker-led test-and-treat outreach models for infectious diseases? (Examples, HIV, hepatitis, COVID-19?)
      1. If yes, which communities utilize this type of model the most?
      2. If not, why do you think that is the case?
   3. What would be key factors in designing and implementing a community health worker led model for viral hepatitis and cancer care for Michigan AAPI communities?
      1. Facilitating factors?
      2. Barriers?
      3. What are people’s thoughts on this type of outreach model to reach Michigan AAPI communities?
         1. How do you think this type of model will be received by Michigan AAPI communities? Why?
5. What are your thoughts on engaging community leaders in the Michigan AAPI communities to enhance viral hepatitis B and C screening and link to care efforts?
   1. What knowledge do you believe Michigan AAPI communities have about viral hepatitis B and C?
      1. Prompts:
         1. Testing recommendations?
         2. Link between liver disease and liver cancer?
         3. Available treatments?
         4. Prevention strategies with harm reduction and HBV vaccination?
6. What are your thoughts on how to engage communities? Can community leaders in the Michigan AAPI communities play a role to enhance liver cancer screening and link to care efforts?
   1. Prompts:
      1. Are they aware of liver cancer disproportionately impacting Michigan AAPI communities?
      2. Do you think they can raise awareness about liver cancer? How would this be best accomplished?
      3. Liver cancer screening including an ultrasound every 6 months (with access to radiology) for certain AAPI populations based on age, sex and severity of liver disease. What are ways we could link people to care to increase liver cancer screening?
   2. Do Michigan AAPI leadership see cancer prevention as an issue in their community? And what about hepatitis screening and education? Please explain.
      1. Would the leadership support additional prevention efforts? Please explain.
7. What are your thoughts on engaging patients living with viral hepatitis and/or cancer in the Michigan AAPI community to enhance hepatitis and cancer screening efforts?
   1. Would the community support these efforts? If yes, how? If not, why?
   2. What are the primary obstacles to these types of efforts in your community?
8. What types of sources does the AAPI community rely on to find out about local events (e.g., word of mouth, flyers)?
   1. How do people obtain health information in Michigan AAPI communities?
9. If I wanted to do a health program with your organization/office/church, what are all the things and who are all the people I would need to know?
10. What are ways to improve health in general in Michigan AAPI communities?
