# Supplementary material for: Identifying determinants of viral hepatitis and liver cancer care in Michigan Asian American communities through multilevel engagement
Source: Hepatol Commun. 2025 Sep 5;9(9):e0803. doi: 10.1097/HC9.0000000000000803 (PMC12412735; doi:10.1097/HC9.0000000000000803)
Supplement: Supplementary file 3 [file hc9-9-e0803-s003.docx]

**Supplement 3: Assigning Barriers and Facilitators to NIMHD and TPB Frameworks, Where X’s Indicate Discussion**

| **Viral hepatitis and liver cancer determinants in Michigan Burmese, Bangladeshi, and Chinese communities** | **Bangladeshi** | **Chinese** | **Burmese** | **External Stakeholders** |
| --- | --- | --- | --- | --- |
| **BARRIERS** |  |  |  |  |
| **NIMHD Theme: Physical/Built Environment** |  |  |  |  |
| Transportation |  | X | X | X |
| Work priorities | X | X | X | X |
| Fear of lost wages or losing employment with taking time off for health matters | X | X | X |  |
| Lack of funding and resources to support outreach/education programs | X | X | X | X |
| **NIMHD Theme: Healthcare System** |  |  |  |  |
| Lack of insurance and costs of care with insurance | X | X | X | X |
| Lack of knowledge about insurance coverage |  | X | X |  |
| Policy changes and restriction (prior authorization) |  |  |  | X |
| Low health literacy and education level | X | X | X | X |
| Symptom-driven health-seeking behavior | X | X | X |  |
| Preference to seek follow-up care and treatment in their home country, lack of belief in Western medicines |  | X | X |  |
| Difficulties of arranging follow-up care after screening, long wait time |  | X |  |  |
| **NIMHD Theme: Sociocultural Environment** |  |  |  |  |
| Inequality of healthcare access in home country and the U.S. |  |  | X |  |
| Medical mistrust, fear of discrimination |  |  | X | X |
| Racism or other prior negative experiences with healthcare providers/systems |  |  | X |  |
| Seniors in the community lack support, are more isolated, and less likely to follow-up |  | X | X |  |
| Non-English language | X | X | X | X |
| Community divided by multiple dialects |  |  | X |  |
| **TPB Theme: Knowledge** |  |  |  |  |
| Lack of knowledge of viral hepatitis and liver cancer | X | X | X | X |
| Misinformation of viral hepatitis and liver cancer | X | X | X | X |
| Lack of communication and information from healthcare providers | X |  |  |  |
| **TPB Theme: Health Behaviors, Attitudes, and Beliefs** |  |  |  |  |
| Stigma | X | X | X |  |
| Fear of testing and what positive results lead to | X | X |  |  |
| Privacy concerns |  | X | X |  |
| Fear of communicating with strangers |  | X | X |  |
| **FACILITATORS** |  |  |  |  |
| **NIMHD Theme: Physical/Built Environment** |  |  |  |  |
| Multisite community-based organizations | X | X |  | X |
| Community partnerships | X | X | X | X |
| Community already has an interest in cancer screening programs |  | X |  |  |
| Combine viral hepatitis screening with other community events | X | X | X | X |
| Convenience of testing |  | X |  | X |
| **NIMHD Theme: Healthcare System** |  |  |  |  |
| Supportive service through healthcare systems, incentives for health engagement, free cancer screening |  |  | X | X |
| **NIMHD Theme: Sociocultural Environment** |  |  |  |  |
| Trust in community leaders, involved in outreach | X | X | X | X |
| Family support | X | X |  |  |
| Language translation services |  | X | X | X |
| Use of social media to connect community | X | X | X | X |
| **TPB Theme: Knowledge** |  |  |  |  |
| Prior viral hepatitis community work, programs, or plans | X | X |  | X |
| In person and virtual health education programs | X | X | X | X |
| **TPB Theme: Health Behaviors, Attitudes, and Beliefs** |  |  |  |  |
| Personal stories/connection to viral hepatitis or liver cancer | X |  | X | X |
| Positive health messaging |  | X |  |  |
| Reassurance regarding privacy of testing results |  | X |  | X |
